# The oxidant-antioxidant imbalance was involved in the pathogenesis of chronic rhinosinusitis with nasal polyps

**DOI:** 10.3389/fimmu.2024.1380846

**Published:** 2024-05-02

**Authors:** Jing Zhou, Jiao Zhou, Ruowu Liu, Yafeng Liu, Juan Meng, Qiao Wen, Yirui Luo, Shixi Liu, Huabin Li, Luo Ba, Jintao Du

**Affiliations:** ^1^ Department of Otolaryngology-Head & Neck Surgery, West China Hospital, Sichuan University, Chengdu, China; ^2^ Upper Respiratory Tract Laboratory of Department of Otolaryngology-Head and Neck Surgery, West China Hospital, Sichuan University, Chengdu, China; ^3^ Department of Medicine and Engineering Interdisciplinary Research Laboratory of Nursing & Materials, West China Hospital, Sichuan University, Chengdu, China; ^4^ Department of Otolaryngology, People’s Hospital of Tibet Autonomous Region, Lhasa, China; ^5^ Department of Otolaryngology, Head and Neck Surgery, Affiliated Eye, Ear, Nose and Throat Hospital, Fudan University, Shanghai, China

**Keywords:** CRSwNP, oxidative stress, nasal epithelial cells, macrophages, Nrf2

## Abstract

**Background:**

Although oxidative stress is involved in the pathophysiological process of chronic rhinosinusitis with nasal polyps (CRSwNP), the specific underlying mechanism is still unclear. Whether antioxidant therapy can treat CRSwNP needs further investigation.

**Methods:**

Immunohistochemistry, immunofluorescence, western blotting and quantitative polymerase chain reaction (qPCR) analyses were performed to detect the distribution and expression of oxidants and antioxidants in nasal polyp tissues. qPCR revealed correlations between oxidase, antioxidant enzymes and inflammatory cytokine levels in CRSwNP patients. Human nasal epithelial cells (HNEpCs) and primary macrophages were cultured to track the cellular origin of oxidative stress in nasal polyps(NPs) and to determine whether crocin can reduce cellular inflammation by increasing the cellular antioxidant capacity.

**Results:**

The expression of NOS2, NOX1, HO-1 and SOD2 was increased in nasal epithelial cells and macrophages derived from nasal polyp tissue. Oxidase levels were positively correlated with those of inflammatory cytokines (IL-5 and IL-6). Conversely, the levels of antioxidant enzymes were negatively correlated with those of IL-13 and IFN-γ. Crocin inhibited M1 and M2 macrophage polarization as well as the expression of NOS2 and NOX1 and improved the antioxidant capacity of M2 macrophages. Moreover, crocin enhanced the ability of antioxidants to reduce inflammation via the KEAP1/NRF2/HO-1 pathway in HNEpCs treated with SEB or LPS. Additionally, we observed the antioxidant and anti-inflammatory effects of crocin in nasal explants.

**Conclusion:**

Oxidative stress plays an important role in the development of CRSwNP by promoting various types of inflammation. The oxidative stress of nasal polyps comes from epithelial cells and macrophages. Antioxidant therapy may be a promising strategy for treating CRSwNP.

## 1 Introduction

Chronic rhinosinusitis with nasal polyps (CRSwNP) is a common chronic inflammatory disease that results in impaired quality of life and a heavy economic burden (1, 2). CRSwNP is generally divided into eosinophilic CRSwNP (ECRSwNP) and noneosinophilic CRSwNP (nECRSwNP) based on the degree of eosinophil infiltration (3–5). Caucasian CRSwNP patients tend to exhibit greater eosinophilic inflammation than Asian patients, while the prevalence of ECRSwNP is increasing in Asian countries (6, 7). Currently, the etiology of CRSwNP has not been fully elucidated. It has been reported that epithelial cells play an important role, as do immune and inflammatory cells, such as macrophages, T and B lymphocytes, group 2 innate lymphoid cells (ILC2s), eosinophils, neutrophils, and mast cells (1, 8, 9). However, the detailed pathogenesis of CRSwNP, especially ECRSwNP, is still unclear, which poses challenges in disease treatment. Therefore, further insight into the pathogenesis of CRSwNP is critical for its management.

Oxidative stress is an imbalance between the production of free radicals and their elimination by an organism’s antioxidant system (10, 11). The most important sources of free radicals are mitochondria, NADPH oxidase (NOX), nitric oxide synthase (NOS) and xanthine oxidase (XO) (12). In contrast, there are many antioxidants in cells that can prevent the production of free radicals or eliminate them quickly, including glutathione (GSH), superoxide dismutase (SOD), catalase (CAT), heme oxygenase (HO) and glutathione reductase (GR) (10). Recently, oxidative stress has been shown to be involved in the development of CRSwNP (13–15). It was reported that patients with nasal polyps show an increase in oxidants and a decrease in antioxidants (16–18). Malgorzata and Yu found that there was no significant difference in SOD activity between patients with nasal polyps and healthy subjects, although HO-1 mRNA and protein expression were significantly increased in nasal polyp tissues compared with healthy control tissues (19, 20). Moreover, we speculate that oxidative stress may have different effects on different types of nasal polyps, but this requires further investigation.

An increasing number of studies have shown that antioxidant therapeutic strategies are beneficial for the treatment of diabetes, coronary artery disease and neurological disorders (21, 22). Crocin, which is an antioxidant, is widely used to treat Alzheimer’s disease and cardiovascular diseases (23, 24) by inhibiting the occurrence of oxidative stress. However, the effect of crocin on CRSwNP is unknown. Herein, we attempted to reveal the status of oxidative stress in nasal polyps and the main cells in which oxidative stress occurs. Furthermore, we explored the therapeutic effect of crocin on nasal polyps to identify potential therapeutic targets for nasal polyps.

## 2 Materials and methods

### 2.1 Patients and tissue samples

Nasal polyp samples from CRSwNP patients were obtained through functional endoscopic sinus surgery at the Department of Otolaryngology Head & Neck Surgery, West China Hospital, Sichuan University. Turbinate tissues were collected from patients who were undergoing endoscopic skull base surgery or septoplasty. We diagnosed CRSwNP according to EPOS 2020, and ECRSwNP met the requirement of more than 10 eosinophils in each high power field (HFP) of three random fields. Otherwise, it is nECRSwNP (3). Patients who were younger than 18 years old, treated with corticosteroids, antihistamines, or antibiotics before surgery and patients with antrochoanal polyps, autoimmune disease, ciliary dysfunction, fungal rhinosinusitis, and inverted papilloma were excluded. The skin prick test was applied to evaluate the atopic status of the patient. Patient comorbidities and basic demographic data were documented preoperatively. This study was approved by the Medical Ethics Committee of the West China Hospital of Sichuan University, and informed consent was obtained prior to the study. The clinical characteristics of the control and CRSwNP groups are shown in **Supplementary Table 1**.

### 2.2 Immunohistochemistry

Tissues were embedded in paraffin following ethyl alcohol dehydration and then sectioned to 4 μm thickness. Immunohistochemistry (IHC) staining was performed with a universal detection kit (PV-6000, ZSGB-Bio, Beijing, China) and DAB Detection System (ZLI-9017, ZSGB-Bio, Beijing, China). The sections were air-dried overnight at 37°C, followed by deparaffinization, hydration, antigen retrieval and endogenous peroxidase removal. Then, sections were incubated with primary antibodies against NOS2 (1:400, ABclonal, Wuhan, China), 3-nitrotyrosine (3-NT) (1:200, Abcam, Cambridge, UK), HO-1 (1:400, ABclonal, Wuhan, China) and SOD2 (1:400, ABclonal, Wuhan, China)at 4°C overnight, and incubated with enzyme-labeled sheep anti-mouse/rabbit IgG polymer at 37°C for 20 min. Next, slides were stained with freshly prepared DAB, counterstained with hematoxylin and finally imaged under a 400-fold microscope.

### 2.3 Western blotting

SDS−PAGE gels were used to separate the proteins, after which the proteins were transferred to PVDF membranes (Millipore, MA, USA). Membranes were blocked in TBST containing 5% nonfat milk for 1 h at room temperature (RT). Then, membranes were incubated with primary antibodies against NOS2 (1:1000, ABclonal, Wuhan, China), NOX1 (1:1000, ABclonal, Wuhan, China), HO-1 (1:1000, ABclonal, Wuhan, China), SOD2 (1:1000, ABclonal, Wuhan, China), Kelch-like ECH-associated protein 1 (KEAP1) (1:1000, Proteintech, Wuhan, China), nuclear factor κB (NF-κB) P65 (1:1000, Cell Signaling Technology, Danvers, USA), GAPDH (1:10000, Proteintech, Wuhan, China), Histone H3 (1:4000, HUABIO, Hangzhou, China) and Tublin-α (1:10000, abcam, USA) at 4 °C overnight followed by incubation with secondary antibody (1:5000, Proteintech, Wuhan, China) for 1 h at RT. Finally, membranes were visualized by NcmECL Ultra (NCM Biotech, Shanghai, China).

### 2.4 Total RNA extraction and real-time quantitative PCR

Animal Total RNA Isolation Kit (FOREGENE, Chengdu, China) and Cell Total RNA Isolation Kit (FOREGENE, Chengdu, China) were used to extract total RNA from nasal tissues and cell, respectively. Total RNA was reverse transcribed into cDNA using HiScript III All-in-one RT SuperMix Perfect for qPCR (Vazyme Biotech, Nanjing, China) according to the manufacturer’s protocols. qPCR was achieved with synthetic primers and Taq pro Universal SYBR qPCR Master Mix (Vazyme Biotech, Nanjing, China). The related primers used are shown in **Supplementary Table 2**.

### 2.5 Immunofluorescence

Paraffin sections were prepared with deparaffinization, hydration, and antigen retrieval, and HNEpC were fixed in 4% paraformaldehyde for 15 min at RT. Both tissues and cells were permeabilized in PBS supplemented with 0.1% Triton X-100. Then, 5% BSA supplemented with 0.01% Triton X-100 was used to block nonspecific binding for 2 h at RT. Sections were incubated overnight at 4°C with primary antibodies against CD68 (1:2000, Proteintech, Wuhan, China), NOS2 (1:400, ABclonal, Wuhan, China), HO-1 (1:400, ABclonal, Wuhan, China), SOD2 (1:400, ABclonal, Wuhan, China) and nuclear factor E2-related factor 2 (NRF2) (1:200, Proteintech, Wuhan, China), and cells were incubated with primary antibodies against NRF2 and NF-κB P65 (1:400, Cell Signaling Technology, Danvers, USA), followed by incubation with fluorophore-conjugated secondary antibodies. Finally, sealing tablets containing DAPI were added to the slides, which were later covered by cover glasses.

### 2.6 Culture and polarization of macrophages

Peripheral blood mononuclear cells (PBMCs) were isolated from healthy controls by Ficoll-Paque PREMIUM density gradient (GE Healthcare, USA) centrifugation. The CD14+ monocytes were separated by positive magnetic selection (Miltenyi Biotec, Germany). PBMCs were labeled with CD14 MicroBeads for 20 min at 4°C in the dark. Then, the cells were washed and immediately sorted on a MACS Separator (Miltenyi Biotec, Germany) to obtain CD14+ monocytes.

The CD14+ monocytes were cultured in serum-free RPMI-1640 medium for 2 h. After the cells adhered to the wall, the medium was changed to serum-free medium (LONZA, Switzerland) containing 20 ng/ml macrophage colony-stimulating factor (M-CSF) (PeproTech, USA). The medium was changed every 3 days and supplemented with M-CSF. The cells were induced to M0 macrophages (M0) after one week. To polarize into M1 macrophages (M1), cells were treated with 100 ng/ml LPS and 20 ng/ml interferon-γ (IFN-γ) (Novoprotein, Shanghai, China) for 24 h, and cells were stimulated with 20 ng/ml interleukin (IL)-4 (Novoprotein, Shanghai, China) for 24 h to polarize into M2 macrophages (M2).

### 2.7 Culture of HNEpC

The HNEpC line was kindly donated by the First Affiliated Hospital of Sun Yat-sen University. The culture medium used for HNEpC was RPMI-1640 medium containing 10% fetal bovine serum, 100 U/mL penicillin, and 100 μg/mL streptomycin (Gibco, Paisley, UK). The cells were stimulated by either lipopolysaccharide (LPS) (Sigma−Aldrich, USA) or staphylococcal enterotoxin B (SEB) (Toxin Technology, Sarasota, FL, USA) with or without crocin (target mol, USA) when they reached 70-80% confluency.

### 2.8 Culture of nasal polyp explants

Fresh nasal polyp tissues were obtained from CRSwNP patients during surgery, washed with RPMI-1640 medium three times, and then cut into smaller pieces weighing approximately 40 mg each. Then, the tissue was passed through a mesh (pore size 0.9 mm^2^) to acquire tissue fragments, which were resuspended in a 12-well plate and cultured in RPMI-1640 medium containing 10% fetal bovine serum, 100 U/mL penicillin, and 100 μg/mL streptomycin. Explants were treated with either SEB or LPS for 24 h with or without crocin.

### 2.9 Statistical analysis

Statistical analyses were performed by using GraphPad Prism 7.0 (GraphPad Software, San Diego, USA). The Kruskal−Wallis test was used for comparisons between multiple groups in nasal tissues and the Spearman correlation coefficient was applied to determine variable relationships in nasal polyp tissues. Cell culture data are presented as the mean ± standard deviation (M ± SD) and were analyzed using one-way analysis of variance (ANOVA). Asterisks indicate statistical significance (*p<0.05, **p<0.01, ***p<0.001, ****p<0.0001).

## 3 Results

### 3.1 The expression and distribution of oxidases in different nasal tissues

**Figure 1:**
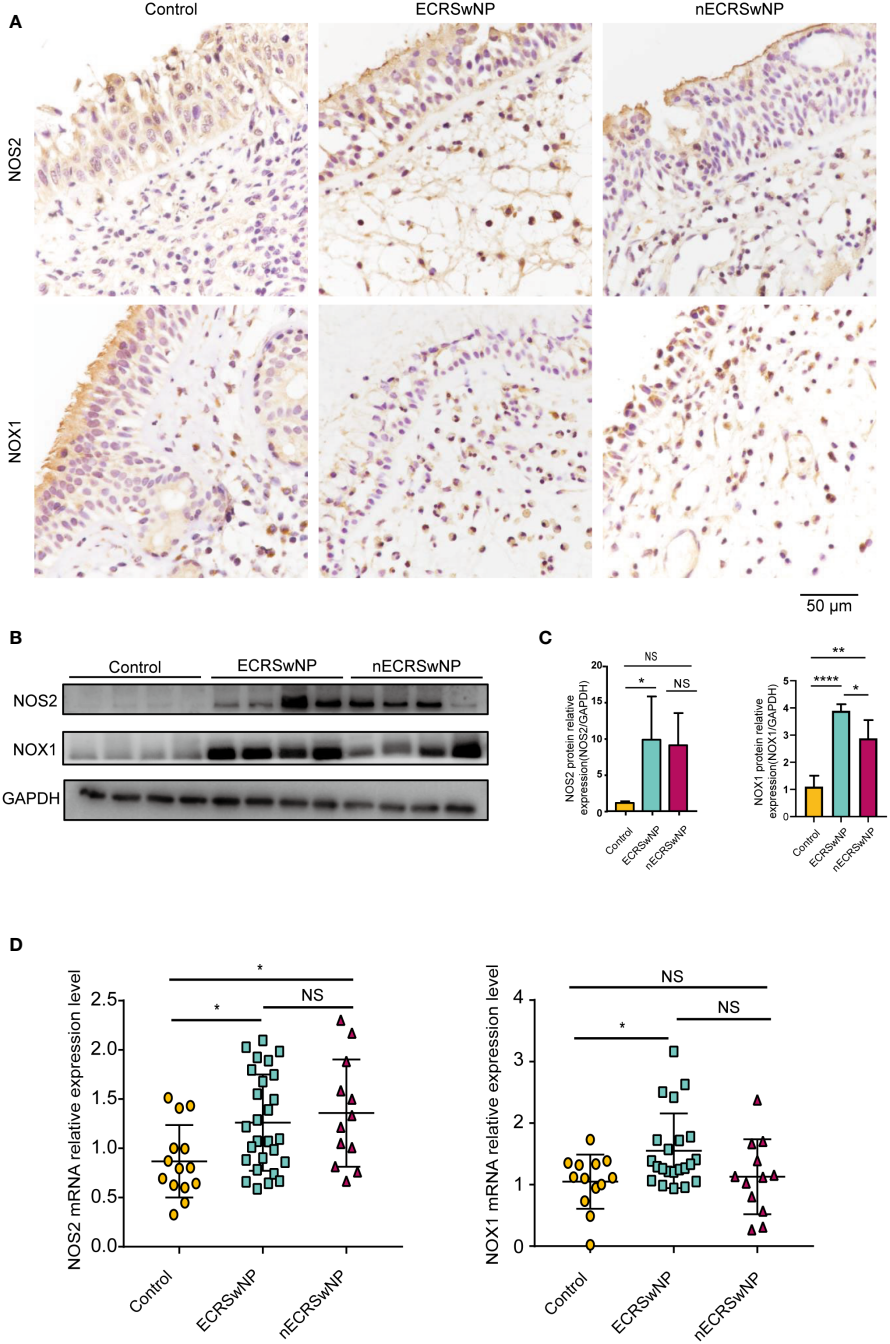
The expression of oxidase was increased in ECRSwNP compared with control. **(A)** The location of NOS2 and NOX1 was detected in control, ECRSwNP and nECRSwNP by IHC staining. **(B)** NOS2 and NOX1 protein expression was determined by Western blotting in control subjects, ECRSwNP patients and nECRSwNP patients. **(C)** Relative protein levels of NOS2 and NOX1 were normalized to GAPDH in control (n=4), ECRSwNP (n=4) and nECRSwNP (n=4). **(D)** Real-time quantitative PCR results for NOS2 in control subjects (n=14), ECRSwNP (n=27) and nECRSwNP (n=12) and for NOX1 in control subjects (n=13), ECRSwNP (n=22) and nECRSwNP (n=12). The Kruskal−Wallis test was used for comparisons among multiple groups. *p<0.05, **p<0.01, ****p<0.0001. NS, Not significant.

Patients were divided into the control group, ECRSwNP group and nECRSwNP group according to the number of infiltrated eosinophils in the tissue sections (**Supplementary Figure 1**). Furthermore, oxidase expression and distribution were determined by IHC, and the results showed that NOS2 was more highly expressed in the epithelial and submucosal cells of the NP than in those of the control mucosa and that NOX1 was more strongly expressed in the submucosal cells of nasal tissues (**Figure 1A**). Increased levels of the NOS2 and NOX1 proteins were detected in samples from ECRSwNP patients compared with control samples (**Figures 1B, C**). Moreover, the mRNA expression of NOS2 and NOX1 was significantly greater in ECRSwNP patients than in control subjects (**Figure 1D**). As a product of protein oxidation, 3-nitrotyrosine (3-NT) is a marker of oxidative damage (25). 3-NT expression was greater in the epithelial cells and submucosal cells of NPs than in those of UPs from control subjects (**Supplementary Figure 2**). Collectively, our data revealed an increase in oxidative stress in CRSwNP patients, especially in ECRSwNP patients.

### 3.2 The expression and distribution of antioxidant enzymes in different nasal tissues

**Figure 2:**
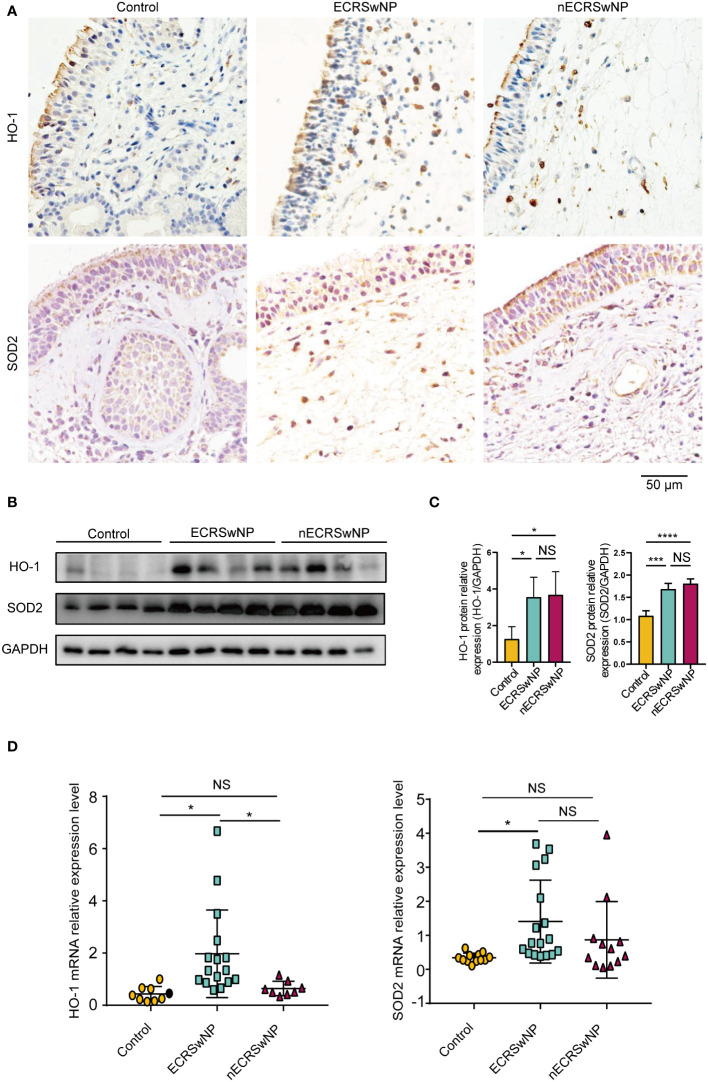
The expression of antioxidases was increased in ECRSwNP compared with control. **(A)** The location of HO-1 and SOD2 in control tissues and nasal polyps as detected by IHC. **(B)** Western blotting analyses of HO-1 and SOD2 protein levels in control subjects, ECRSwNP patients and nECRSwNP patients. **(C)** HO-1 and SOD2 relative protein levels were normalized to GAPDH in control subjects (n=4), ECRSwNP patients (n=4) and nECRSwNP patients (n=4). **(D)** qPCR results for HO-1 in control subjects (n=9), ECRSwNP (n=16) and nECRSwNP (n=8) and for SOD2 in control subjects (n=12), ECRSwNP (n=17) and nECRSwNP (n=12). The Kruskal−Wallis test was used for comparisons among multiple groups. *p<0.05, ***p <0.001, ****p <0.0001.. NS, Not significant.

Immunostaining showed that HO-1 was mainly expressed in the submucosal cells of NPs from CRSwNP patients (**Figure 2A**). SOD2 was distributed primarily in epithelial cells, submucosal cells and submucosal glands in nasal tissues and was increased in the submucosal cells of CRSwNP NPs compared with control NPs (**Figure 2A**). In contrast to previous studies (20, 26), we found that HO-1 and SOD2 protein levels were significantly greater in CRSwNP patients than in controls (**Figures 2B, C**). In addition, we found that HO-1 mRNA levels were significantly greater in ECRSwNP patients than in nECRSwNP patients and control subjects, and SOD2 mRNA expression was significantly greater in ECRSwNP patients than in control subjects (**Figure 2D**). In summary, we observed that the expression of antioxidant enzymes increased with increasing oxidative stress in ECRSwNP patients.

### 3.3 Correlations between oxidase and antioxidant enzyme expression and inflammatory cytokine levels in CRSwNP patients

**Figure 3:**
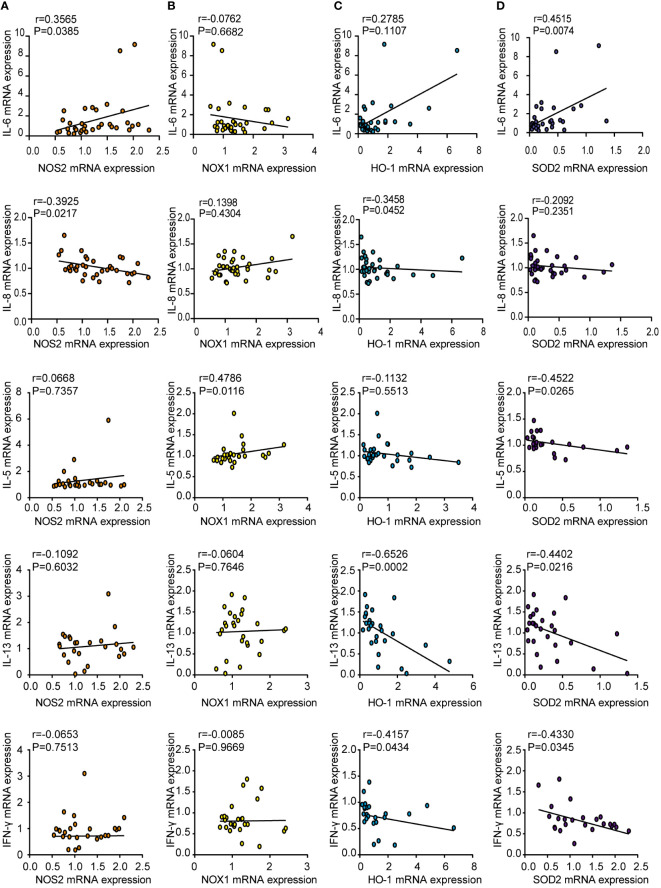
Correlations between oxidase and antioxidase expression and inflammatory cytokines in CRSwNP. Spearman analyses on the correlation of IL-6, IL-8, IL-5, IL-13, IFN-γ mRNA levels and the mRNA levels of NOS2 **(A)**, NOX1 **(B)**, HO-1 **(C)**, SOD2 **(D)** in nasal polyps tissue from CRSwNP.

To elucidate the role of oxidases and antioxidant enzymes in CRSwNP, we examined the relationship between these enzymes and inflammatory cytokines, such as IL-6, IL-8, IL-5, IL-13 and IFN-γ, in NPs from patients with CRSwNP. NOS2 mRNA levels were positively correlated with IL-6 mRNA expression (**Figure 3A**). The mRNA levels of IL-5 were positively correlated with NOX1 mRNA expression (**Figure 3B**). In addition, the mRNA expression of HO-1 had a negative relationship with that of IL-8, IL-13 and IFN-γ (**Figure 3C**). We also found that SOD2 expression was negatively correlated with IL-5, IL-13, and IFN-γ levels (**Figure 3D**). In general, oxidase levels may be positively correlated with inflammatory cytokine expression, while antioxidant enzyme levels can be negatively correlated with cytokine expression. We speculate that the increase in oxidative stress may lead to an increase in inflammatory cytokine levels, whereas increased antioxidant enzymes may inhibit inflammation.

### 3.4 In CRSwNP, macrophages are involved in oxidation and antioxidation

**Figure 4:**
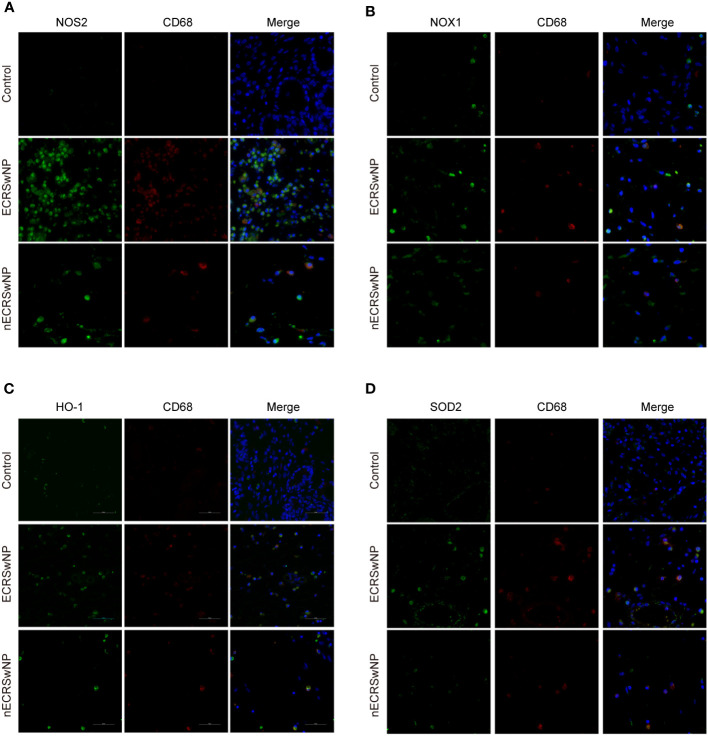
Coexpression of oxidase, antioxidase and CD68 in human nasal tissues. Representative immunostaining photomicrographs show colocalization of CD68 with NOS2 **(A)**, NOX1 **(B)**, HO-1 **(C)**, and SOD2 **(D)** in control, ECRSwNP and nCRSwNP tissue samples. Green staining indicates NOS2, NOX1, HO-1 and SOD2. Red and blue staining indicates CD68 and DAPI (nuclei), respectively.

Previous studies have shown that macrophages are the main cellular source of reactive oxygen species (ROS)/reactive nitrogen species (RNS) in the lungs and can produce NO through NOS2 (27). We investigated the expression of oxidase and antioxidant enzymes in macrophages labeled with CD68 by dual immunofluorescence staining of nasal tissue sections. Our results showed that NOS2 (green), NOX1 (green), HO-1 (green), and SOD2 (green) could be coexpressed with CD68 (red) in nasal tissue from patients with CRSwNP (**Figures 4A–D**). Therefore, macrophages are likely the main source of free radicals.

### 3.5 The damage of antioxidant enzymes is related to M2 macrophage polarization

**Figure 5:**
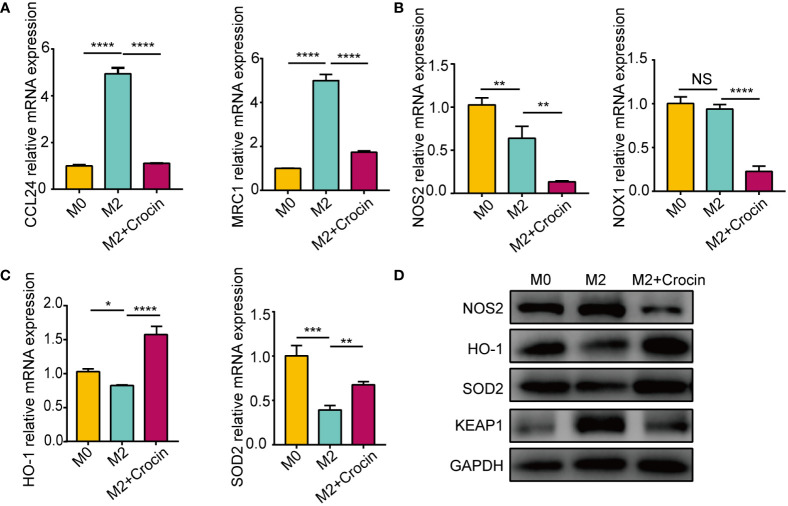
The damage of antioxidant enzymes is related to M2 polarization. M0 were pretreated with IL-4 (20 ng/ml) with or without crocin (20 µM) for 24 h. **(A-C)** qPCR was used to detect the mRNA expression of CCL24, MRC1, NOS2, NOX1, HO-1 and SOD2. **(D)** Western blotting was used to evaluate the protein expression of NOS2, HO-1, SOD2 and KEAP1. Data were obtained in three independent experiments. One-way ANOVA was used to analyze the differences between multiple groups. *p<0.05, **p<0.01, ***p<0.001, ****p<0.0001.. NS, Not significant.

Real-time quantitative PCR showed that IL-4 promoted M2 macrophage polarization by increasing the mRNA expression of CCL24 and MRC1 (**Figure 5A**). The expression of NOS2 and NOX1 was not increased in M2 macrophage (**Figures 5B, D**) but was increased in M1 macrophages (**Supplementary Figure 3B**), which means that M1 activation induced oxidative stress. M2 polarization suppressed the expression of the antioxidant enzymes HO-1 and SOD2 (**Figures 5C, D**). Interestingly, crocin strongly inhibited the expression of M2 markers (**Figure 5A**), increased HO-1 and SOD2 expression (**Figures 5C, D**) and decreased NOS2, NOX1 and KEAP1 expression in M2 macrophages (**Figures 5B, D**). Moreover, crocin decreased M1 macrophages polarization (**Supplementary Figure 3A**). In summary, we speculated that M1 polarization results in increased oxidative stress, but M2 polarization impairs antioxidant capacity. In addition, crocin simultaneously inhibited M1 polarization by directly reducing the level of oxidative stress and inhibited M2 polarization by improving the antioxidant capacity.

### 3.6 Crocin treatment attenuates oxidative injury and inflammation in HNEpCs via the KEAP1-NRF2/HO-1 pathway

**Figure 6:**
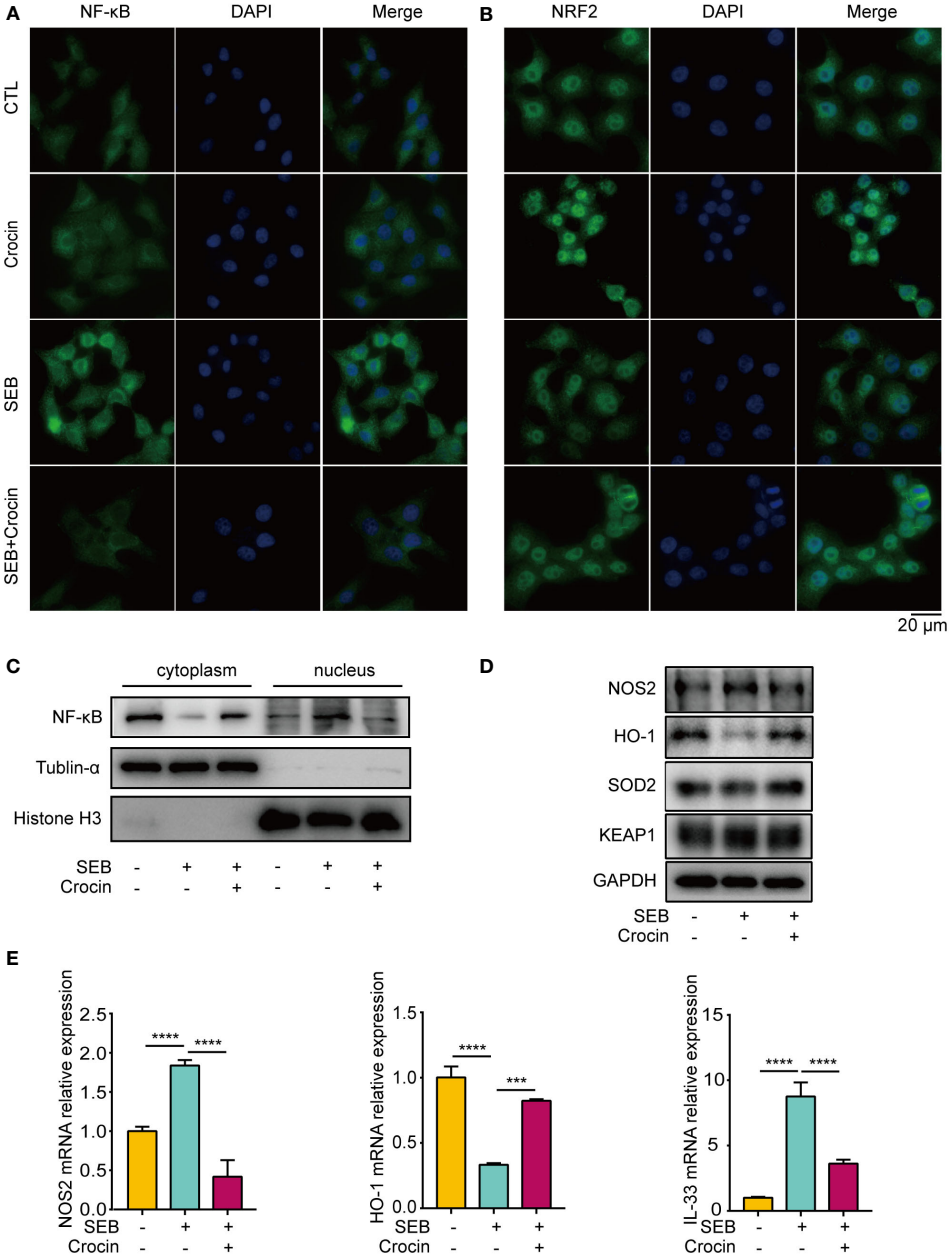
Crocin treatment attenuates oxidative injury and inflammation in HNEpC. **(A, B)** HNEpC were pretreated with or without crocin (20 µM) for 24 h and incubated with or without SEB (1 μg/mL) for 2 h. Cells were visualized by immunostaining with anti-NF-κB (green) and anti-NRF2 (green) antibodies. Nuclei were stained with DAPI (blue). **(C)** HNEpC were incubated with SEB (1 μg/mL) with or without crocin (20 µM) for 24 h followed by nucleocytoplasmic separation. **(D)** Western blotting showed changes in NOS2, HO-1, SOD2 and KEAP1 protein levels. **(E)** qPCR showed changes in NOS2, HO-1 and IL-33 mRNA levels. Data were obtained in three independent experiments. One-way ANOVA was used to analyze the differences between multiple groups. ***p<0.001, ****p<0.0001.

As oxidase and antioxidant enzymes are expressed in the epithelial cells of NPs from patients with CRSwNP, we explored the relationship between epithelial cells and oxidative stress. Our results showed that the nuclear localization of NF-κB was increased in the SEB treatment group (**Figures 6A, C**). In addition, the mRNA and protein levels of NOS2 were increased in the SEB-treated group (**Figures 6D, E**), and the level of IL-33 was increased in the SEB-treated group (**Figure 6E**). These findings indicated that SEB treatment activated oxidative injury and cell inflammation in HNEpCs. Crocin has been proven to ameliorate cardiotoxicity via the KEAP1-NRF2/HO-1 pathway (28). Our results revealed that NRF2 translocated into the nucleus of HNEpCs treated with crocin (**Figure 6B**). SEB treatment decreased HO-1 protein and mRNA levels (**Figures 6D, E**). Interestingly, SEB treatment did not change SOD2 protein levels (**Figure 6D**). Furthermore, crocin induced NF-κB translocation from the nucleus to the cytoplasm (**Figure 6A**), and the expression of NOS2, IL-33 was inhibited by crocin (**Figures 6D, E**). These results provide further evidence that crocin attenuates oxidative injury and inflammation in HNEpCs through the KEAP1-NRF2/HO-1 pathway. We also found that LPS treatment activated inflammation and crocin reversed this effect (**Supplementary Figure 4**).

### 3.7 Crocin reduces the inflammation of nasal polyp explants stimulated with SEB or LPS

**Figure 7:**
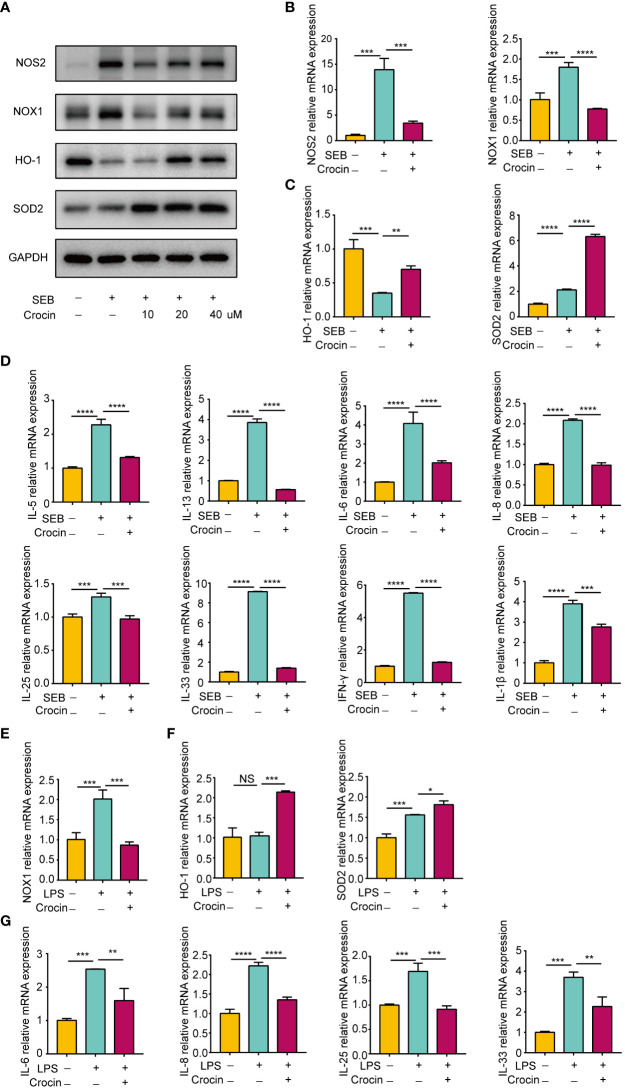
Crocin reduces the inflammation of nasal polyp explants induced by SEB or LPS. **(A–D)** Nasal polyp explants were incubated with SEB (500 ng/ml) with or without crocin (20 µM) for 24 h. **(A)** Protein expression of NOS2, NOX1, HO-1, SOD2 was assessed by Western blotting. **(B)** mRNA expression of NOS2 and NOX1 was measured by qPCR. **(C)** mRNA expression of HO-1 and SOD2 were measured by qPCR. **(D)** Significant downregulation of IL-6, IL-8, IL-5, IL-13, IL-25, IL-33, IL-1β and IFN-γ mRNA levels after crocin treatment. **(E, F)** Nasal polyp explants were treated with LPS (100 ng/ml) with or without crocin (20 µM) for 24 h. **(E)** mRNA expression of NOX1, HO-1 and SOD2 was measured by qPCR. **(F)** mRNA expression of IL-6, IL-8, IL-25 and IL-33 was measured by qPCR. One-way ANOVA was used to analyze the differences between multiple groups. *p<0.05, **p<0.01, ***p<0.001, ****p<0.0001.. NS, Not significant.

Nasal polyp explants have the advantage of replicating the *in situ* mucosal environment, so we confirmed the antioxidant effect of crocin on nasal polyps based on *in vitro* explant models. SEB significantly increased NOS2 and NOX1 expression (**Figures 7A, B**). Moreover, HO-1 expression was decreased, while SOD2 expression was elevated by SEB (**Figures 7A, C**). In contrast, crocin decreased NOS2 expression and NOX1 expression (**Figures 7A, B**) and increased the expression of HO-1 and SOD2 (**Figures 7A, C**). SEB also increased IL-5, IL-13, IL-6, IL-8, IL-25, IL-33, IFN-γ and IL-1β mRNA expression, and crocin treatment reversed this effect (**Figure 7D**). In addition, LPS stimulation increased NOX1 mRNA expression as well as that of SOD2, IL-6, IL-8, IL-25, and IL-33 (**Figures 7E, F**). Crocin reduced NOX1, IL-6, IL-8, IL-25, and IL-33 mRNA expression but increased HO-1 and SOD2 expression (**Figures 7E, F**). Together, these results indicated that crocin exerts an anti-inflammatory effect on nasal polyp explants by improving the antioxidant capacity of cells.

## 4 Discussion

Oxidative stress has been shown to be involved in a wide range of diseases, including chronic obstructive pulmonary disease, Alzheimer’s disease, cancer and cardiovascular and metabolic diseases (21, 29, 30). To protect against oxidative stress damage, organisms have evolved defense mechanisms based on antioxidant enzymes that neutralize oxidants and repair oxidative injury. Therefore, these defense mechanisms are the targets of disease treatment and prevention, and agents that enhance antioxidative defenses are the main strategies of antioxidant therapy (21). For example, N-acetylcysteine (NAC) can treat nephropathy by supplementing with GSH (31). Recently, oxidative stress was also shown to play crucial roles in the development of CRSwNP (32). In the present study, we assessed the oxidative status and antioxidative defense in CRSwNP patients and observed an imbalance between oxidase and antioxidant enzyme levels, particularly in patients with ECRSwNP. We further found that the accumulation of oxidants was positively correlated with the levels of inflammatory cytokines. Thus, improving the antioxidant capacity to reduce oxidation and inflammation may be a potential strategy for the treatment of CRSwNP.

Oxidation and antioxidation are dynamically balanced, and an increase in the oxidant level will promote an increase in the antioxidants by negative feedback, thus eliminating excessive ROS and reducing oxidative stress. Consistent with previous studies (33–35), we found that compared with that in the control group, the expression of NOS2 and NOX1 was increased markedly in ECRSwNP patients. Notably, for the first time, we reported an increase in 3-NT expression in nasal polyps. Moreover, the antioxidant level also increased, mainly manifested as an increase in the levels of antioxidant enzymes such as SOD2 and HO-1. However, our results are slightly different from those of previous reports. Ono et al. (26) found that the mRNA and activity of SOD2 were decreased in patients with CRSwNP, especially in patients with ECRSwNP, compared with healthy controls. In addition, Yu et al. (20) reported that the mRNA expression of HO-1 in nECRSwNP patients was significantly greater than that in ECRSwNP patients. Therefore, we speculate that oxidative status may be related to the unclear development of nasal polyps and drug interference during treatment. Further research revealed that the expression of these oxidation-related genes were positively correlated with the levels of inflammatory factors, while the expression of antioxidant genes were negatively correlated with inflammatory factor levels. These studies showed that there is an obvious imbalance between oxidation and antioxidation in nasal polyps, which could be involved in the inflammatory reaction in nasal polyps.

Previous studies have shown that nasal epithelial cells, macrophages, neutrophils, eosinophils and basophils are the sites of oxidative stress in CRSwNP (13, 14, 16). In our study, NOS2 and NOX1 were expressed in epithelial and subcutaneous cells, such as NOS2^+^ macrophages, as determined by immunofluorescence. As reported, epithelial cells are frequently exposed to exogenous oxidants such as pathogens, allergens, cigarette smoke, diesel fuel, and ozone, which can lead to the development of oxidative stress (36). We observed an imbalance between the oxidation and antioxidation of HNEpCs stimulated by LPS and SEB, which was characterized by a decrease in HO-1 expression and an increase in NOS2 expression. Moreover, the translocation of NF-κB to the nucleus was also determined by immunofluorescence. It has been reported that macrophages can be polarized into M1-like macrophages (M1) and M2-like macrophages (M2) (37). Consistent with previous research results (38), M1 macrophages exhibit obvious oxidative stress and inflammatory responses, including increased expression of NOS2 and NOX1, decreased expression of HO-1 and increased expression of IL-6 and tumor necrosis factor α (TNF-α). However, the antioxidant enzyme SOD2 in M1 macrophages was also increased, consistent with the findings of Paulina Tokarz’s research (39), which suggested that the increased expression of SOD2 protected macrophages from LPS-induced damage. In contrast, decreased expression of NOS2 and NOX1 as well as antioxidant enzymes (HO-1 and SOD2) were detected in polarized M2 macrophages. These results in macrophages indicated that oxidative stress is mainly produced by M1-like macrophages. In summary, epithelial cells and macrophages are important sources of oxidative stress in CRSwNP, while the increased expression of antioxidant enzymes in these cells reduces oxidative stress and inflammation to a certain degree as a feedback mechanism.

Redox balance is regulated by the KEAP1/NRF2/ARE signaling pathway which is the main pathway involved in the protection against oxidative stress in different diseases (40–42). Under stress conditions, ROS can modify the cysteine residues of KEAP1, resulting in inactivation of the E3 ubiquitin ligase and accumulation of NRF2 (43). NRF2 then translocates into the nucleus and binds to antioxidant response elements (AREs) to initiate the transcription of antioxidant genes, including HO-1, NQO1 and SOD (44). In this study, we found increased expression of both oxidases and the antioxidant enzymes SOD2 and HO-1 in nasal polyps, indicating the activation of the KEAP1/NRF2 signaling pathway in response to stress. However, the increase in antioxidant enzymes failed to offset the accumulation of oxidants, leading to increased oxidative stress in nasal tissue. Previous studies have confirmed that the KEAP1/NRF2 system could be an important therapeutic target for various diseases, including inflammatory diseases (40), diabetes (41), and neurodegenerative diseases (42). For example, mangiferin and panaxydol can alleviate allergic rhinitis (AR) and LPS-induced lung inflammation through the KEAP1-NRF2/HO-1 pathway, respectively (45).

Crocin, which is extracted from saffron, has been proven to have antioxidant and anti-inflammatory properties (46). Several studies have confirmed that crocin has significant therapeutic effects on diabetes (47), cancer (48), and cardiovascular disease (49). Our results showed that crocin treatment alleviated inflammation and oxidative stress in HNEpCs stimulated with LPS and SEB. Crocin strongly inhibited the expression of NF-κB p65 and its translocation into the nucleus (50) but significantly activated NRF2 (28). Interestingly, the expression of SOD2 was not affected by crocin, which showed that crocin inhibited LPS- and SEB-induced inflammation though KEAP1-NRF2/HO-1 signaling in HNEpCs. Previous studies have shown that crocin alleviates coronary atherosclerosis and titanium particle-induced inflammation by inducing M2 macrophage polarization (51, 52). Unlike previous studies, we observed that crocin inhibited the expression of MRC1 and CCL24 in M2 macrophages and inhibited the expression of IL-6, NOS2 and TNF-α in M1 macrophages, which indicated that crocin may inhibit M1 and M2 macrophage polarization. Furthermore, crocin upregulated the expression of HO-1 and SOD2 in M2 macrophages but did not upregulate the expression of HO-1 and SOD2 in M1 macrophages. In addition, KEAP1 protein levels were decreased in the M2 group treated with crocin. These results suggested that crocin could achieve antioxidant and anti-inflammatory effects through the KEAP1/NRF2 pathway in M2 macrophages, while it may directly reduce the inflammatory response induced by M1 macrophages in other ways. Because M2 macrophages has been demonstrated to play an important role in persistent inflammation in CRSwNP, particularly in ECRSwNP (53, 54), crocin may have an anti-inflammatory effect on CRSwNP due to its ability to increase antioxidant enzyme expression in M2 macrophages. As crocin is a natural product with low toxicity, it has great application prospects. Overall, treatment with antioxidants might be a potential strategy for CRSwNP management.

## 5 Conclusions

Our results revealed that an imbalance between oxidants and antioxidants could be involved in the development of chronic rhinosinusitis with nasal polyps. Nasal epithelial cells and macrophages are the main cellular sources of oxidative stress in CRSwNP. Antioxidant treatment with crocin might be a potential strategy for the treatment of CRSwNP through the KEAP1/NRF2/HO-1 pathway.

## Data availability statement

The raw data supporting the conclusions of this article will be made available by the authors, without undue reservation.

## Ethics statement

The studies involving humans were approved by Biomedical Research Ethics Committee of West China Hospital of Sichuan University. The studies were conducted in accordance with the local legislation and institutional requirements. The participants provided their written informed consent to participate in this study.

## Author contributions

JinZ: Conceptualization, Methodology, Writing – review & editing, Data curation, Formal analysis, Investigation, Software, Writing – original draft. JiaZ: Conceptualization, Project administration, Supervision, Writing – review & editing, Data curation, Formal analysis, Investigation. RL: Data curation, Formal analysis, Writing – review & editing. YL: Resources, Supervision, Writing – review & editing. JM: Supervision, Writing – review & editing, Funding acquisition. QW: Data curation, Formal analysis, Software, Visualization, Writing – review & editing. YL: Conceptualization, Funding acquisition, Project administration, Supervision, Writing – review & editing. SL: Conceptualization, Funding acquisition, Project administration, Resources, Supervision, Validation, Writing – review & editing. HL: Supervision, Validation, Writing – review & editing. LB: Funding acquisition, Methodology, Supervision, Writing – review & editing. JD: Funding acquisition, Methodology, Supervision, Writing – review & editing, Conceptualization, Project administration, Validation.
