# Supplementary material for: The oxidant-antioxidant imbalance was involved in the pathogenesis of chronic rhinosinusitis with nasal polyps
Source: Front Immunol. 2024 May 2;15:1380846. doi: 10.3389/fimmu.2024.1380846 (PMC11096511; doi:10.3389/fimmu.2024.1380846)
Supplement: Supplementary file 1 [file DataSheet_1.docx]

| **Supplemental Table 1.** Clinical characteristics of control, ECRSwNP and nECRSwNP | | | | |
| --- | --- | --- | --- | --- |
|  | Control | ECRSwNP | nECRSwNP | *p* Value |
| Sex (male:female ratio) | 14 (12:2) | 27 (16:11) | 12 (10:2) | - |
| Age, median (range) | 31(18-55) | 40(13-67) | 50(22-60) | - |
| Allergic rhinitis (n) | 0 | 6 | 1 | - |
| Asthma (n) | 0 | 10 | 1 | - |
| SPT^*^ (n) | 1 | 3 | 1 | - |
| Serum eosinophil (%) | 2.96±1.58 | 7.26±3.29 | 4.99±2.52 | <0.001 |
| eosinophil counts | 0.16±0.08 | 0.44±0.22 | 0.33±0.17 | <0.001 |
| number (n)/ HPF^#^ | 0.3±0.5 | 30.3±10.3 | 2.4±10.3 | <0.001 |

^*^SPT: skin prick test

^#^HPF: high powered field

**Supplemental Table 2. The primer sequences used in qPCR**

| Gene | 5′ ------ Forward ------ 3′ | 5′------ Reverse ------ 3′ |
| --- | --- | --- |
| *GAPDH* | CATCAAGAAGGTGGTGAAGCA | TCAAAGGTGGAGGAGTGGGT |
| NOS2 | CCTCAAGTCTTATTTCCTCAACGTT | CCGATCAATCCAGGGTGCTA |
| NOX1 | CTGCTTCCTGTGTGTCGCAA | AGGCAGATCATATAGGCCACC |
| HO-1 | AAGACTGCGTTCCTGCTCAAC | AAAGCCCTACAGCAACTGTCG |
| SOD2 | GGCCTACGTGAACAACCTGA | TTCCAGCAACTCCCCTTTGG |
| IL-6 | CAACCTGAACCTTCCAAAGATG | ACCTCAAACTCCAAAAGACCAG |
| IL-8 | AAGGTGCAGTTTTGCCAAGG | CAACCCTCTGCACCCAGTTT |
| IL-5 | ATCTTTCAGGGAATAGGCACAC | CCGTCTTTCTTCTCCACACTTT |
| IL-13 | CCTCATGGCGCTTTTGTTGAC | TCTGGTTCTGGGTGATGTTGA |
| IFN-γ | TCGGTAACTGACTTGAATGTCCA | TCGCTTCCCTGTTTTAGCTGC |
| IL-33 | AGGAGAGAAACCACCAAAAGG | CTGGACCCCTGATATACCAAAG |
| IL-25 | GGCTGTACCGTGTTTCCTTAG | CTTCATGGCAAGTGGTTGTAC |
| IL-1β | AGCTACGAATCTCCGACCAC | CGTTATCCCATGTGTCGAAGAA |
| TNF-α | CAGCCTCTTCTCCTTCCTGA | ACCCACTCCTCCACCTTTGAC |
| CCL24 | CTACGGGCTCTGTGGTC | GGTTTGGTTGCCAGGATA |
| MRC1 | ACACCAAAACCTGAGCCAAC | CCACCCATCTTCAGTAACTGGT |

**
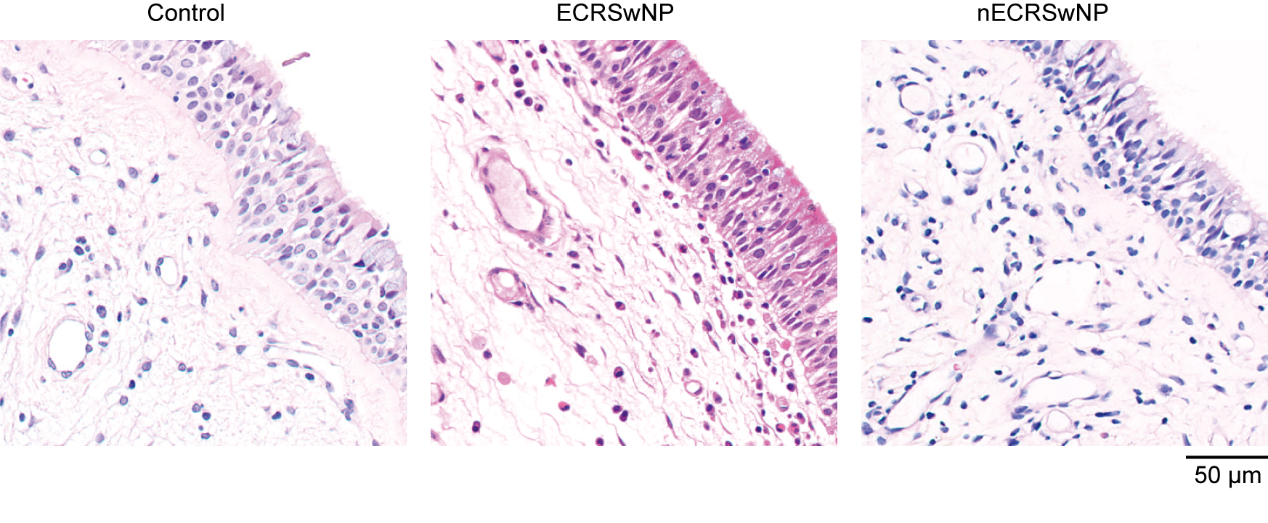
Supplementary Figure 1**. Types of nasal polyps according to number of eosinophils.

Hematoxylin-eosin staining of healthy controls, patients with nECRSwNP and patients with ECRSwNP is shown.

**Supplementary Figure 2**. The location of 3-NT was detected in control, ECRSwNP and nECRSwNP by immunofluorescence


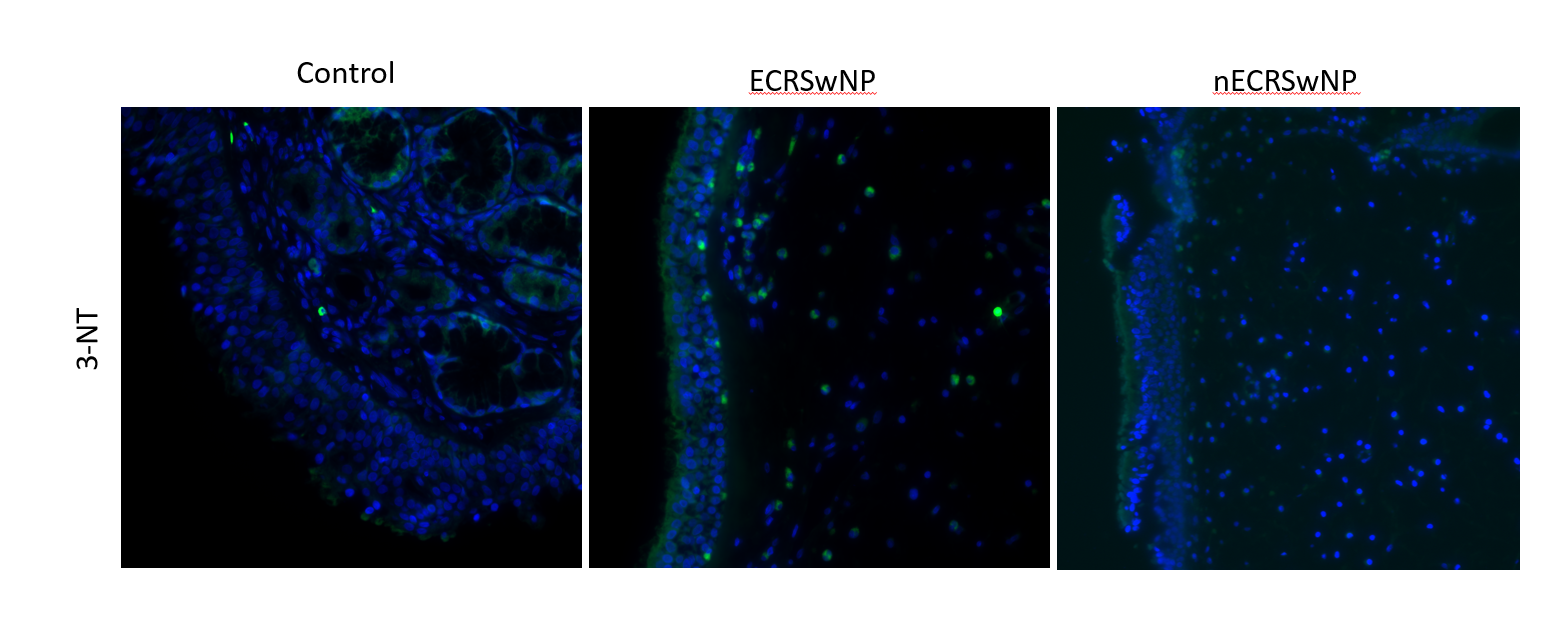


**
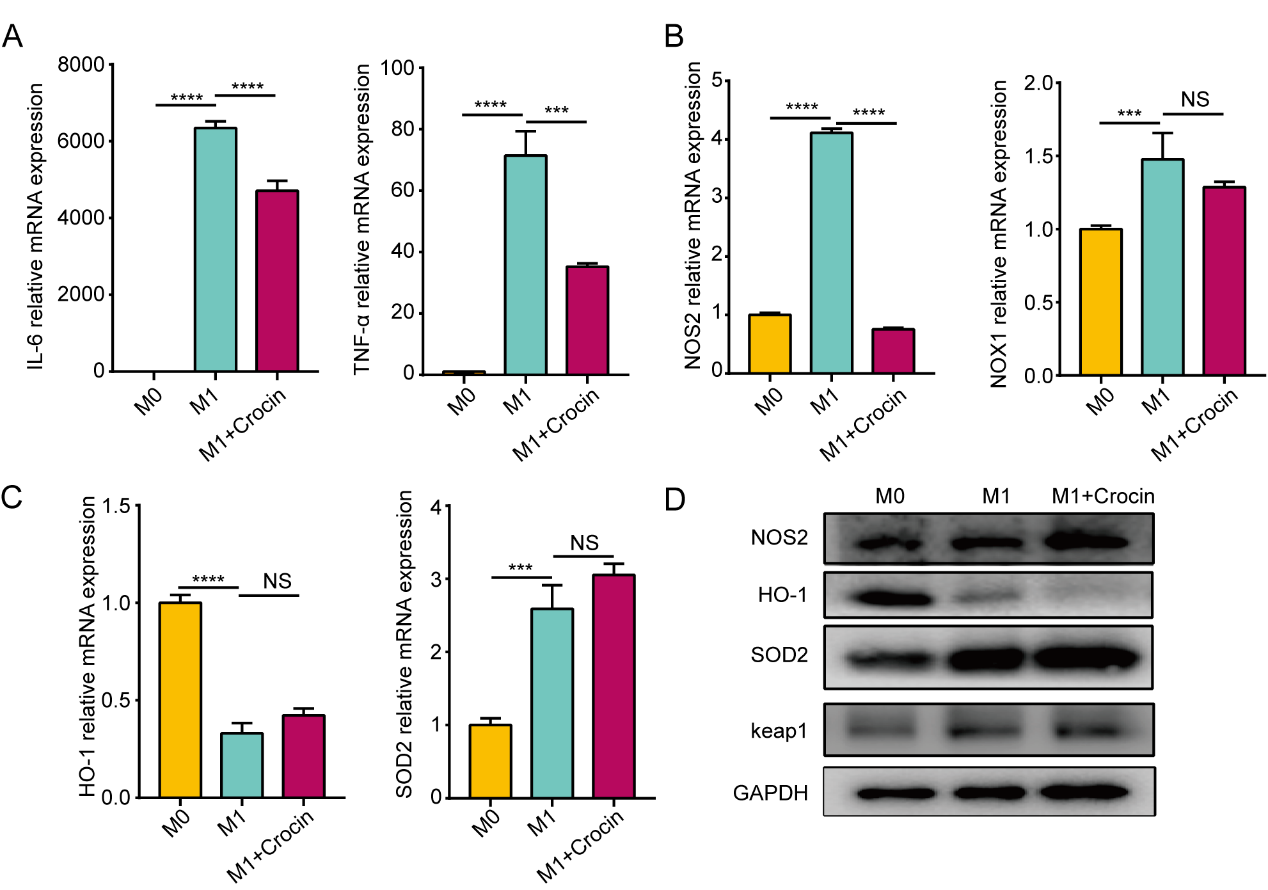
Supplementary Figure 3.** Crocin inhibited M1 macrophage polarization.

M0 were pretreated with LPS (100ng/ml) and IFN-γ (20ng/ml) with or without crocin (20uM) for 24 h. (A-C) qPCR was used to detect mRNA expression of IL-6, TNF-α, NOS2, NOX1, HO-1 and SOD2. (D)Western blotting was used to evaluate protein expression of NOS2, HO-1, SOD2 and KEAP1.

**Supplementary Figure 4.**

**
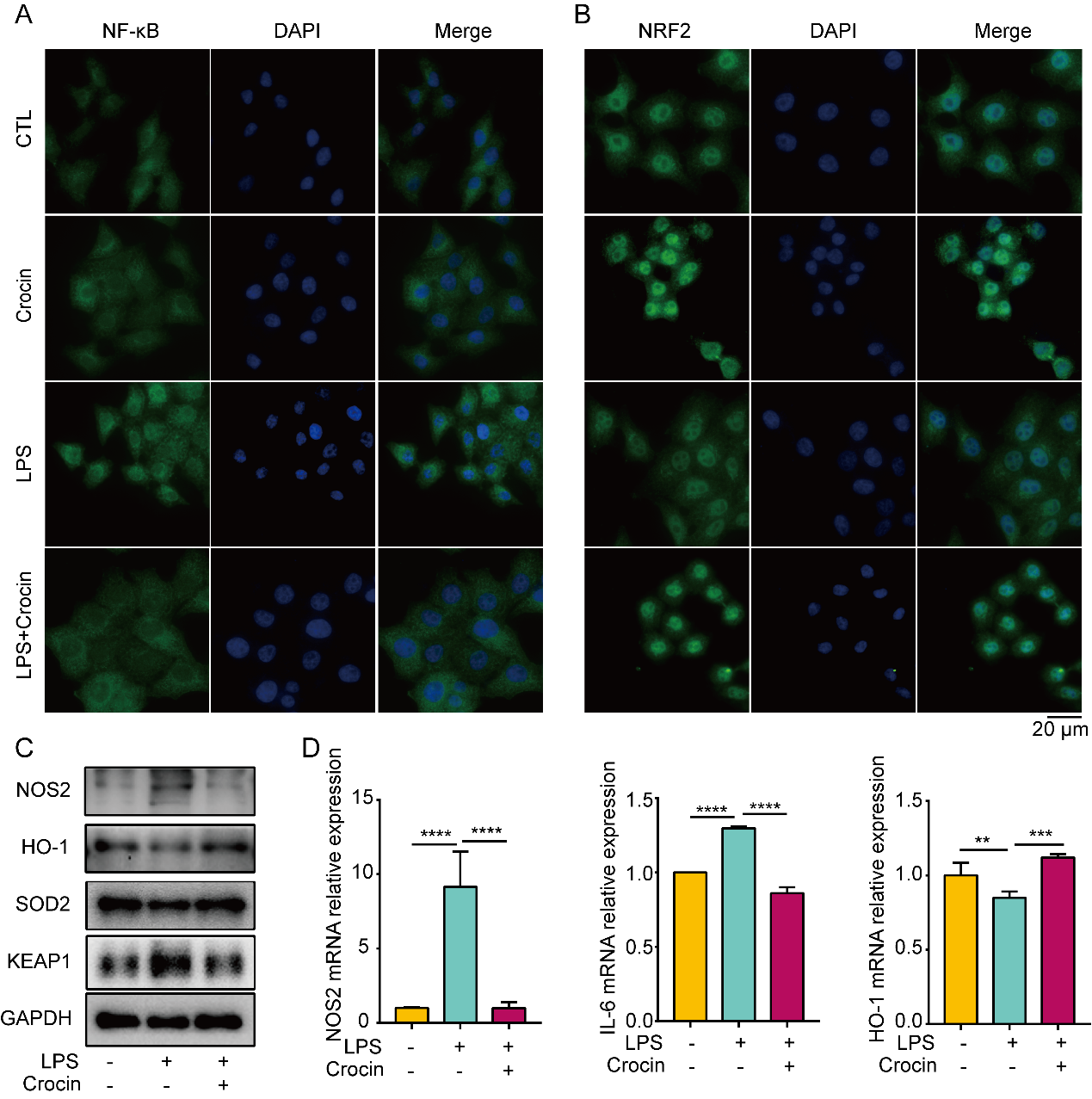
**

(A, B) HNEpC were pretreated with or without crocin (20 µM) for 24 h and incubated with or without LPS (1 μg/mL) for 2 h. Cells were visualized by immunostaining with anti-NF-κB (green) and anti-NRF2 (green) antibodies. Nuclei were stained with DAPI (blue). (C) Western blotting showed changes in NOS2, HO-1, SOD2 and KEAP1 protein levels. (D) qPCR showed changes in NOS2, IL-6 and HO-1 mRNA levels. Data were obtained in three independent experiments. One-way ANOVA was used to analyze the differences between multiple groups. ***p*<0.01, ****p*<0.001, *****p*<0.0001.
